# SGLT2 inhibition via dapagliflozin improves generalized vascular dysfunction and alters the gut microbiota in type 2 diabetic mice

**DOI:** 10.1186/s12933-018-0708-x

**Published:** 2018-04-27

**Authors:** Dustin M. Lee, Micah L. Battson, Dillon K. Jarrell, Shuofei Hou, Kayl E. Ecton, Tiffany L. Weir, Christopher L. Gentile

**Affiliations:** Department of Food Science and Human Nutrition, Colorado State University, Fort Collins, CO 80523 USA

**Keywords:** SGLT2, Dapagliflozin, Vascular function, Arterial stiffness, Aortic pulse wave velocity, Type 2 diabetes, Gut microbiota

## Abstract

**Background:**

Type 2 diabetes (T2D) is associated with generalized vascular dysfunction characterized by increases in large artery stiffness, endothelial dysfunction, and vascular smooth muscle dysfunction. Sodium glucose cotransporter 2 inhibitors (SGLT2i) represent the most recently approved class of oral medications for the treatment of T2D, and have been shown to reduce cardiovascular and overall mortality. Although it is currently unclear how SGLT2i decrease cardiovascular risk, an improvement in vascular function is one potential mechanism. The aim of the current study was to examine if dapagliflozin, a widely prescribed STLT2i, improves generalized vascular dysfunction in type 2 diabetic mice. In light of several studies demonstrating a bi-directional relation between orally ingested medications and the gut microbiota, a secondary aim was to determine the effects of dapagliflozin on the gut microbiota.

**Methods:**

Male diabetic mice (Db, n = 24) and control littermates (Con; n = 23) were randomized to receive either a standard diet or a standard diet containing dapagliflozin (60 mg dapagliflozin/kg diet; 0.006%) for 8 weeks. Arterial stiffness was assessed by aortic pulse wave velocity; endothelial function and vascular smooth muscle dysfunction were assessed by dilatory responses to acetylcholine and sodium nitroprusside, respectively.

**Results:**

Compared to untreated diabetic mice, diabetic mice treated with dapagliflozin displayed significantly lower arterial stiffness (Db = 469 cm/s vs. Db + dapa = 435 cm/s, p < 0.05), and improvements in endothelial dysfunction (area under the curve [AUC] Db = 57.2 vs. Db + dapa = 117.0, p < 0.05) and vascular smooth muscle dysfunction (AUC, Db = 201.7 vs. Db + dapa = 285.5, p < 0.05). These vascular improvements were accompanied by reductions in hyperglycemia and circulating markers of inflammation. The microbiota of Db and Con mice were distinctly different, and dapagliflozin treatment was associated with minor alterations in gut microbiota composition, particularly in Db mice, although these effects did not conclusively mediate the improvements in vascular function.

**Conclusions:**

Dapagliflozin treatment improves arterial stiffness, endothelial dysfunction and vascular smooth muscle dysfunction, and subtly alters microbiota composition in type 2 diabetic mice. Collectively, the improvements in generalized vascular function may represent an important mechanism underlying the cardiovascular benefits of SGLT2i treatment.

## Background

Type 2 diabetes (T2D) affects nearly 30 million individuals in the US and over 350 million individuals worldwide [1]. Among the myriad health consequences of T2D, cardiovascular disease (CVD) is the most common and critical. Individuals with T2D are more than twice as likely as individuals without diabetes to develop CVD during their lifetime; and CVD is the most common cause of hospitalization and death in people with diabetes, accounting for nearly 70% of diabetes-related fatalities [2]. Although there is a strong correlation between the magnitude of hyperglycemia and CVD event rates among people with diabetes [3, 4], the cardiovascular benefits of successful glucose control are unclear, and there remains considerable uncertainty as to whether current anti-diabetic medications reduce CVD risk [5, 6]. As such, identification of novel anti-diabetic therapies with substantiated CVD risk reduction properties is a top clinical priority.

Sodium glucose cotransporter 2 inhibitors (SGLT2i) represent the most recently approved class of oral medications for the treatment of T2D. Early modeling data predicted that, unlike other classes of antidiabetic medications, SGLT2i may have significant beneficial effects on cardiovascular outcomes [7]. Results from the EMPA-REG OUTCOME trial support these predictive data, and found that SGLT2i in patients with T2D reduced cardiovascular mortality by 38%, sudden death by 31%, and hospitalizations for heart failure by 35% [8]. These data have prompted tremendous interest in identifying the underlying physiological changes that mediate the beneficial cardiovascular effects of SGLT2i [9, 10]. One potential but understudied mechanism that may explain the beneficial CV effects of SGLT2i is an improvement in vascular function. Indeed, vascular dysfunction is a hallmark of T2D and may explain much of the heightened CVD risk in people with diabetes [11, 12]. Among the various features of vascular dysfunction, three components in particular contribute to diabetes-related CVD: (1) arterial stiffness, (2) endothelial dysfunction and (3) vascular smooth muscle dysfunction. Arterial stiffness and endothelial dysfunction are well characterized features of T2D, and both precede clinical manifestations of CVD, and are independent predictors of future cardiovascular events in people with diabetes [12, 13]. Vascular smooth muscle dysfunction, as determined by reductions in endothelium-independent dilation (EID), has received considerably less focus in regards to its prognostic potential. However, numerous studies have demonstrated that reductions in EID are commonly observed in T2D [14, 15] and predict cardiovascular complications and mortality [16, 17]. Importantly, few studies have assessed the effects of SGLT2i on vascular dysfunction in people with diabetes. Among the existing studies, variable results have been reported, and no studies have comprehensively examined the effects of chronic SGLT2i on vascular function in type 2 diabetes [18–21]. In light of these data, it is conceivable that the cardio-protective effects of SGLT2i treatment are mediated by improvements in generalized vascular dysfunction.

The gut microbiota has emerged as a critical regular of human physiology and disease processes [22], and data from our laboratory [23] and elsewhere [24] indicate that the gut microbiota is an important regulator of vascular function. Previous studies have also shown that there is a bi-directional interaction between various orally ingested (non-antibiotic) medications and the gut microbiota (i.e. the microbiota affects medication activity; certain medications can affect microbiota composition). For example, various drug classes have been shown to profoundly alter gut microbiota composition [25, 26]; and the gut microbiota can alter drug absorption and metabolism or mediate some of the physiological effects of T2D medications [25, 27]. Importantly, this bi-direction relation extends to various medications for CVD and T2D whose primary site of action is outside of the intestines. Thus, there is tremendous clinical relevance in determining the interaction between the gut microbiota and SGLT2i, although to date, no studies have done so.

With this background, the purpose of the present study was to examine the effects of dapagliflozin, a selective SGLT2i approved for use in treating T2D, on arterial stiffness, endothelial dysfunction, and vascular smooth muscle cell dysfunction in male T2D mice, and to characterize the effects of SGLT2i on the gut microbiota.

## Methods

### Animals and experimental design

Eight-week old diabetic mice homozygous for a point mutation in the leptin receptor gene (C57BLKS/J-lepr^db^/lepr^db^) and age- and gender-matched heterozygous littermates (C57BLKS/J-lepr^db^/+) were obtained from the Jackson Laboratory (Bar Harbor, ME). Mice were housed in a temperature and humidity controlled environment on a 12 h:12 h light–dark cycle. Prior to initiating experimental procedures, mice were acclimatized to the housing conditions for 2 weeks. All animal procedures were reviewed and approved by the Colorado State University Institutional Animal Care and Use Committee. Following acclimatization, control (n = 23) and diabetic (n = 24) mice were randomized to receive one of two diets for 8 weeks: (1) standard diet (D111122001, Research Diets, Inc., New Brunswick, NJ) consisting of 15% fat, 65% carbohydrate, and 20% protein, or (2) standard diet containing dapagliflozin (Farxiga) (60 mg dapagliflozin/kg diet; 0.006%) (D06081805, Research Diets). This resulted in the following four groups: (1) control mice receiving a standard diet (Con; n = 11), (2) control mice reviving standard diet containing dapagliflozin (Con + dapa; n = 12), (3) diabetic mice receiving standard diet (Db; n = 12) and, (4) diabetic mice receiving standard diet containing dapagliflozin (Db + dapa; n = 12). Mice were cohoused according to treatment group. The Con groups were cohoused 4 mice/cage while the Db groups were cohoused 2 mice/cage given the polyuria accompanied by this phenotype. Mice were allowed free access to food and water for the duration of the 8-week intervention; and body weight and food intake were measured weekly.

### Fasting glucose

Fasting blood glucose was determined after a 6 h fast from tail vein blood using a glucometer (AlphaTRAK, Abbott Laboratories, Abbott Park, IL) at weeks 0, 2, 4, and 8.

### Aortic pulse wave velocity (aPWV)

Large elastic artery stiffness was determined by aPWV at baseline and following the 8-week intervention as previously described [23, 28]. Briefly, mice were anesthetized using 2% isoflurane and oxygen at 2 L/min, placed supine on a heating board with legs secured to ECG electrodes, and maintained at a target heart rate of ~ 450 bpm by adjusting isoflurane concentration. Doppler probes (20 MHz) (Mouse Doppler data acquisition system; Indus Instruments) were placed on the transverse aortic arch and abdominal aorta and the distance between the probes was determined with precision calipers. Pre-ejection time, the time between the R-wave of the ECG and the foot of the Doppler signal, was determined for each site. aPWV was calculated by dividing the distance (cm) between the probes by the difference in pre-ejection times (seconds) of the thoracic and abdominal regions.

### Animal termination and tissue collection

Mice were anaesthetized with isoflurane and euthanized by exsanguination via cardiac puncture. Blood was collected with an EDTA-coated syringe and immediately centrifuged at 1000 rcf for 10 min at 4 °C to obtain plasma. Second-order mesenteric arteries were excised in ice-cold physiologic saline solution (PSS: 0.288 g NaH2PO4, 1.802 g glucose, 0.44 g sodium pyruvate, 20.0 g BSA, 21.48 g NaCl, 0.875 g KCl, 0.7195 g MgSO4 7H20, 13.9 g MOPS sodium salt, and 0.185 g EDTA per liter solution at pH 7.4) and cannulated for vascular reactivity experiments (see below). Adipose tissue depots (subcutaneous, epididymal, and mesenteric depots) were excised and weighed.

### Vascular reactivity

Vascular function was determined as previously described [28, 29]. Briefly, second-order mesenteric arteries were placed in pressure myograph chambers (DMT Inc., Atlanta, GA) containing warm PSS, cannulated onto glass micropipettes and secured with suture. Arteries were equilibrated for 1 h at 37 °C and an intraluminal pressure of 50 mmHg. Arteries were constricted with increasing doses of phenylephrine (PE: 10^−9^–10^−5^ M) followed immediately by a dose–response with endothelium-dependent dilator acetylcholine (ACh: 10^−9^–10^−4^ M). We have previously established that arteries maintain constriction to PE for the duration of experiments and that dilation is not spontaneous. If arteries do spontaneously dilate during the constriction period (i.e. 5–12 min), data are not included. After a washout period, a dose–response to endothelium-independent dilator sodium nitroprusside (SNP: 10^−10^–10^−4^ M) was obtained after pre-constriction to PE (10^−5^ M). Percent dilation was calculated based on the maximal luminal diameter of each artery.

### Circulating inflammatory cytokines

Plasma was analyzed in duplicate on a single plate to determine the concentration of circulating inflammatory markers interleukin [IL]-1β, IL-6, IL-10, IL17, monocyte chemoattractant protein-1 (MCP-1), and chemokine ligand 5 (CCL5) using a multiplex assay (MCYTOMAG-70K; EMD Millipore, Billerica, MA, USA). Intra-assay variability (< 5%) was within the normal limits reported by the manufacturer.

### Intestinal microbiota characterization

Feces were collected fresh from individual animals prior to termination and flash frozen in liquid nitrogen. DNA was extracted using the PureLink Microbiome DNA Purification Kit (A29790, Invitrogen, Carlsbad, CA). The 16 s rRNA gene was amplified for sequencing following the Earth Microbiome Project 16 s protocol utilizing 515F-806R primer set (forward: 5′GTGYCAGCMGCCGCGGTAA 3′; reverse 5′ GGACTACNVGGGTWTCTAAT 3′) [30]. Unique 12 bp error correcting barcodes were included in the construct of the forward primer. Cycling conditions using the Biorad CFX96 thermal cycler were as follows: 94 °C for 3 min and then 35 cycles of 94 °C 45 s, 50 °C 60 s, 72 °C 90 s followed by 72 °C for 10 min. Paired-end sequencing libraries of the V4 region were then constructed by purifying amplicons using AmPure beads and quantifying and pooling equimolar ratios of each sample library. The pooled library was quantified by qPCR and sequenced on an Illumina MiSeq at the next-generation sequencing facility at Colorado State University. Paired-end sequence reads were concatenated and all combined 16 s sequences were filtered, trimmed and processed with the DADA2 (R bioconductor package [31]) implementation included in the open source bioinformatics tool myPhyloDB version 1.2.1 (http://www.myphylodb.org/). Briefly, all primers were removed from each sequence using the open source Python program Cutadapt [32] and sequence variants were inferred using the default pipeline in DADA2. Each sequence variant identified in DADA2 was classified to the closest reference sequence contained in the Green Genes reference database (Vers. 13_5_99) using the usearch_global option (minimum identity of 97%) contained in the open source program VSEARCH [33]. After processing, data were normalized by rarefaction consisting of Laplace smoothing followed by sub-sampling with replacement. Data were rarefied to 12,629 sequence reads with 100 iterations. ANCoVA analysis was conducted in myPhyloDB [34], and MicrobiomeAnalyst [35] was used to calculate alpha diversity scores, Bray–Curtis distances, and LEfSe. The raw sequencing data and associated metadata will be made available upon request.

### Statistics

Data are expressed as mean ± SEM. Statistical analysis was performed using one-way ANOVA with LSD post hoc test (SPSS for Windows, release 11.5.0; SPSS, Chicago, IL, USA). A mixed model ANOVA (within factor, time; between factor, treatment group) was used for variables measured over time (i.e. body weight and blood glucose). A p value of < 0.05 was considered statistically significant. Microbial community α and β-diversity were calculated using Chao1 and Shannon indices (α-diversity) and Bray–Curtis distances (β-diversity) visualized by principle coordinates analysis (PCoA). Non-parametric Mann–Whitney/Kruskil–Wallace tests were used to determine statistical significance of α-diversity measures and permutational MANOVA was used to determine differences in β-diversity. Microbial markers based on differential abundance among the treatment groups were identified using ANCoVA with post hoc Tukey method for multiple comparisons (p < 0.05) and linear discriminate analysis effect size (LEfSe) with an LDA > 1 and q < 0.05 [36]. Pearson’s correlations were conducted between taxa selected as biomarkers in LEfSe and metadata for endothelial-dependent dilation, endothelial-independent dilation, and aortic pulse wave velocity.

## Results

**Fig. 1:**
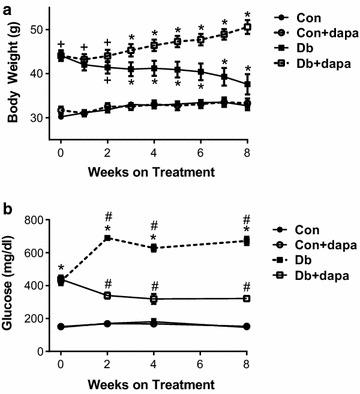
The effects of dapagliflozin on body weight and fasting (6 h) blood glucose. **a** Changes in body weight from weeks 0 to 8; **b** 6 h fasted blood glucose levels from 0 to 8 weeks. Data are expressed as mean ± SEM; *p < 0.05 vs all other groups at the same time point; ^+^p<0.05 vs both Con groups at same time point; ^#^p < 0.05 from week 0 within the same treatment group; n = 11–12/group

Animal characteristics are shown in Table 1. As expected, both groups of diabetic mice (Db and Db + dapa) consumed significantly more food and generally displayed higher body weight (Fig. 1a) and indices of body fat than non-diabetic mice (Con and Con + dapa). Db mice lost weight during the intervention, which is commonly observed in this strain as the diabetic condition advances [37, 38]. Conversely, all other groups significantly gained weight during the intervention. At the end of the 8-week intervention, body weight and indices of body fat were generally higher in Db + dapa compared to all other groups (Table 1). Dapagliflozin treatment did not significantly affect body weight and indices of body fat in Con + dapa mice.

**Table 1:** General and metabolic characteristics

Variable	Con	Con + dapa	Db	Db + dapa
Weekly food intake (g)	20.6 ± 0.3	23.9 ± 0.2	37.6 ± 1.7^#†^	49.8 ± 2.4*
Weight gain (g)	3.8 ± 0.4	2.5 ± 0.4	− 5.2 ± 1.4*	5.4 ± 0.8^‡†^
Subcutaneous Fat (g)	906 ± 70	938 ± 111	3415 ± 339*	4357 ± 204*
Epididymal Fat (g)	1055 ± 63	1117 ± 119	2085 ± 211^#†^	2442 ± 102^#†^
Mesenteric fat (g)	541 ± 35	581 ± 44	957 ± 123*	1331 ± 92*
PVAT (g)	30 ± 2	26 ± 2	36 ± 5	47±5*

Values are mean ± SEM

*PVAT* perivascular adipose tissue; n = 11–12/group

* p < 0.05 vs all other groups

^#^p < 0.05 vs Con

^†^p < 0.05 vs Con + dapa

^‡^p < 0.05 vs Db

At baseline, fasting blood glucose was closely matched between mice of the same genotype randomized to receive dapagliflozin-supplemented or non-supplemented diet; and as expected, fasting blood glucose was markedly higher in both diabetic groups compared to controls (Fig. 1b). Db mice experienced significant worsening of hyperglycemia during the intervention period. In contrast, blood glucose significantly decreased over time in Db + dapa such that levels were significantly lower than (and approximately half of) Db mice at weeks 2, 4 and 8 (Fig. 1b). No effect of time or dapagliflozin treatment were observed in the two Con groups.

**Fig. 2:**
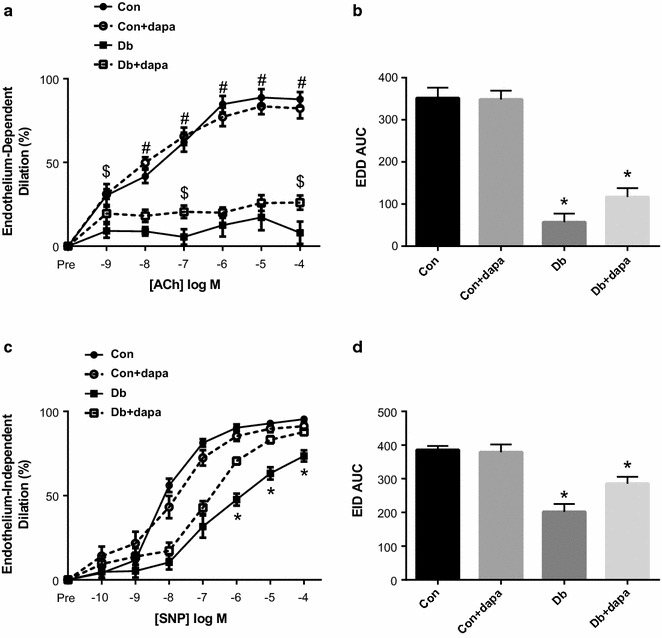
The effects of dapagliflozin on endothelium dependent- and -independent dilation. **a** Endothelium-dependent dilation; **b** area under of the curve for endothelium-dependent dilation (EDD); **c** endothelium-independent dilation; **d** area under of the curve for endothelium-independent dilation (EID). Data are expressed as mean ± SEM; *p < 0.05 vs, all other groups; ^#^p < 0.05 vs both Db groups; ^$^p < 0.05 vs Db group; n = 10–12/group

**Fig. 3:**
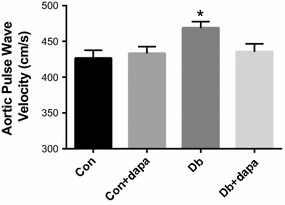
The effects of dapagliflozin on aortic pulse wave velocity (aPWV). aPWV after 8 weeks of dapagliflozin treatment. Data are expressed as mean ± SEM; *p < 0.05 vs all other groups; n = 10–12/group

Endothelium-dependent dilation (EDD) was markedly impaired in both diabetic groups compared to non-diabetic mice. Dapagliflozin treatment improved EDD in Db + dapa mice such that dilation was modestly and significantly increased at several doses of acetylcholine (ACh) (Fig. 2a), and total area under the curve (AUC) was more than doubled (Fig. 2b). Endothelium-independent dilation in Db was significantly impaired and AUC was approximately 50% compared to both Con groups (Fig. 2c, d). Dapagliflozin treatment significantly improved EID in Db + dapa mice such that AUC and dilation to most doses of sodium nitroprusside (SNP) was significantly higher in Db + dapa compared to Db (Fig. 2c, d). The improvement in EID in Db + dapa was such that final dilation to SNP was restored to levels observed in Con + dapa, although AUC remained significantly lower in Db + dapa compared to both non-diabetic groups (Fig. 2d). Neither endothelium-dependent nor -independent dilation differed between Con and Con + dapa, and constriction responses to phenylephrine (PE) did not differ among any groups (data not shown). Aortic stiffness, measured by pulse wave velocity, was significantly higher only in Db, and levels in Db + dapa were similar to Con mice at 8 weeks (Fig. 3).

**Fig. 4:**
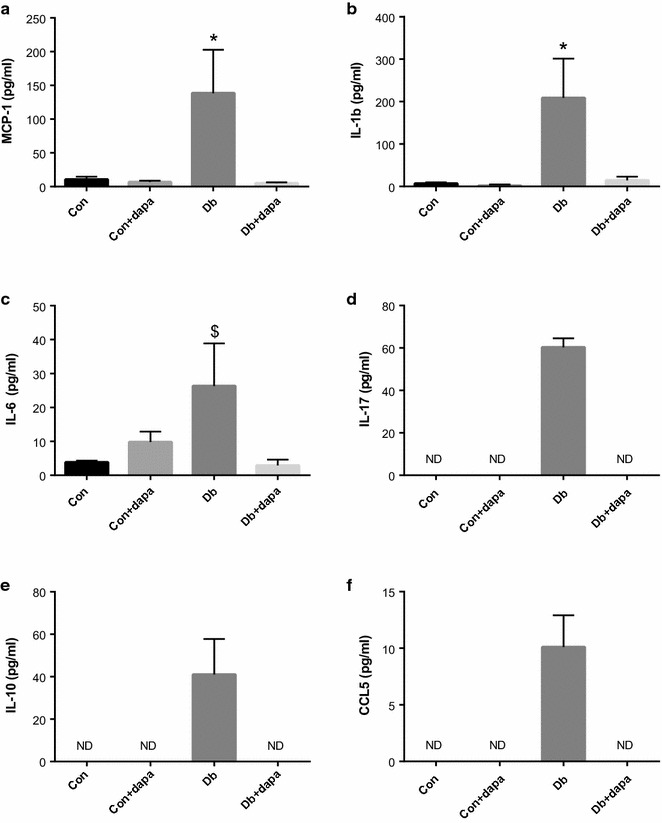
The effects of dapagliflozin on circulating markers of inflammation. **a** Monocyte chemoattractant protein-1 (MCP-1); **b** interleukin-1 beta (IL-1β); **c** interleukin-6 (IL-6); **d** interleukin-17 (IL-17); **e** interleukin-10 (IL-10); **f** chemokine ligand 5 (CCL5). Data are expressed as mean ± SEM; *p < 0.05 vs all other groups; ^$^p < 0.05 vs Con and Db + dapa; n = 3–8/group. *ND* below detectable limits

Given the close association between vascular dysfunction and chronic inflammation [39, 40], we measured several circulating inflammatory markers. MCP-1, IL-1β and IL-6 were all significantly elevated in Db mice and significantly attenuated in Db + dapa mice. IL-17, CCL5, and the anti-inflammatory marker IL-10, were only detected in Db mice and were below detectable limits in all other groups (Fig. 4).

**Fig. 5:**
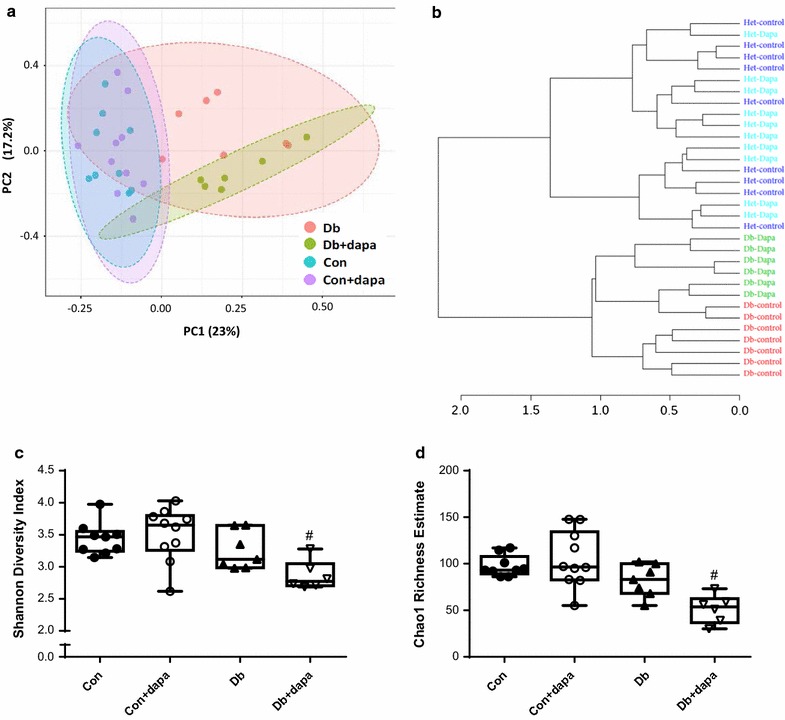
Microbiota characteristics of beta- and alpha-diversity after 8 weeks of treatment with dapagliflozin. **a** PCoA of OTU-level Bray–Curtis; **b** hierarchical clustering of OTU-level Bray–Curtis distances using Ward’s linkage; **c** Chao1 richness estimates; and **d** Shannon diversity measure of fecal samples across treatment groups OTU. Box represents 25th–75th percentiles, median values are represented by boxplot internal line and ranges by whiskers; ^#^p < 0.05 vs both Con groups; n = 6–10/group

**Fig. 6:**
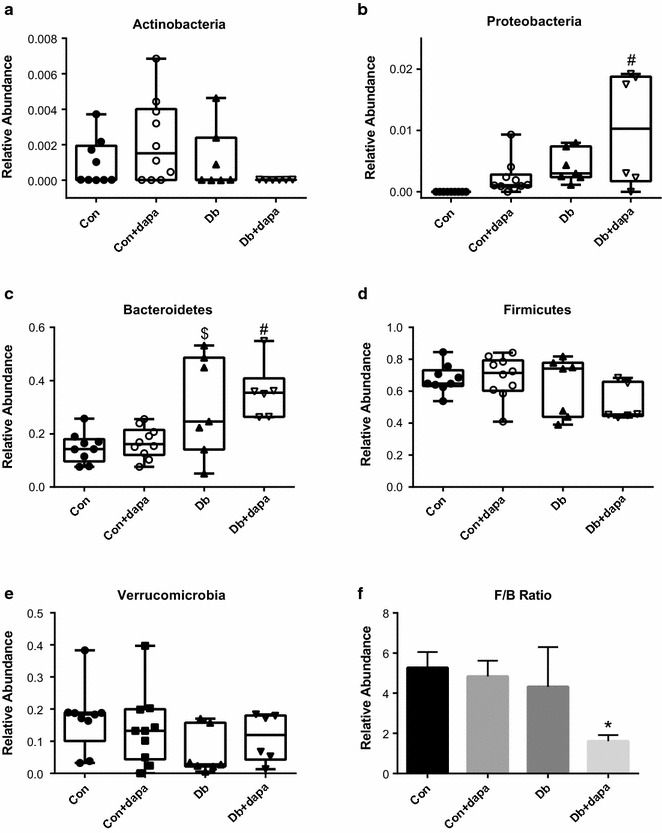
Phyla level characteristics of the microbiota. Relative abundance of **a** Actinobateria; **b** Proteobateria; **c** Bateriodetes; **d** Firmicutes; **e** Verrucomicrobioa; **f** Firmicutes:Bacteriodetes ratio. Box represents 25th–75th percentiles, median values are represented by boxplot internal line and ranges by whiskers; *p < 0.05 vs all other groups, ^#^p < 0.05 vs Con groups, ^$^p < 0.05 vs Con; n = 6–10/group

To examine the effects of SLGT2i on the gut microbiota, we used 16 s sequencing to analyze fecal samples from each mouse. PCoA and hierarchical clustering by Ward’s linkage of these visualizations suggest clustering of microbiota based on mouse genotype (Con vs Db groups), with the Db + dapa animals clustering as a subset within the Db group (Fig. 5a; PCoA: R2 = 0.27488; p < 0.001). Hierarchical clustering of the Db and Db-dapa groups was incomplete, with one sub-cluster containing animals from both groups, suggesting that there were responders and non-responders in this population (Fig. 5b). There was also a subtle, but significant interaction between genotype and dapagliflozin treatment for OTU richness and diversity (Chao1: p = 0.00245; Shannon: p = 0.00832), with significantly reduced richness and diversity in the Db + dapa group compared to Con and Con + dapa groups (Fig. 5c, d). Using ANCoVA for phyla-level taxonomic comparisons among groups, we identified Actinobacteria, Bacteroidetes, Firmicutes, Proteobacteria, and Verrucomicrobia significantly differed among treatment groups (Table 2). Although most of these differences were associated with diabetes status, Bacteroidetes and Proteobacteria also appeared to be influenced by dapagliflozin treatment in a subset of the Db + dapa animals (Fig. 6, Table 2). Calculating the ratio of Firmicutes:Bacteroidetes (F:B) showed that Db + dapa animals had a significantly lower ratio than the other treatment groups (Fig. 6f).

**Table 2:** Phyla level microbiota characteristics

Phyla	Db (all) vs Con (all)	Db (no dapa) vs Con (no dapa)	Db vs Db + dapa	Con + dapa vs Db + dapa
Actinobacteria	0.0431 (0.0007)	NS	NS	NS
Bacteroidetes	0.001 (0.0359)	0.0484 (0.0496)	NS	0.0053 (0.0519)
Firmicutes	0.0319 (0.0443)	NS	NS	NS
Proteobacteria	0.0006 (0.0015)	NS	NS	0.0069 (0.0022)
Verrucomicrobia	0.0494 (0.0333)	NS	NS	NS

Data are expressed as p value (SE), *NS* non-significant; n = 6–10/group. “Db (all) vs Con (all)” indicates interaction between the genotype of Db (diabetic db^+/+^) and Con (heterozygous db^+/−^) mice, irrespective of drug treatment; “no dapa” indicates only those mice not receiving the drug within the genotype

**Fig. 7:**
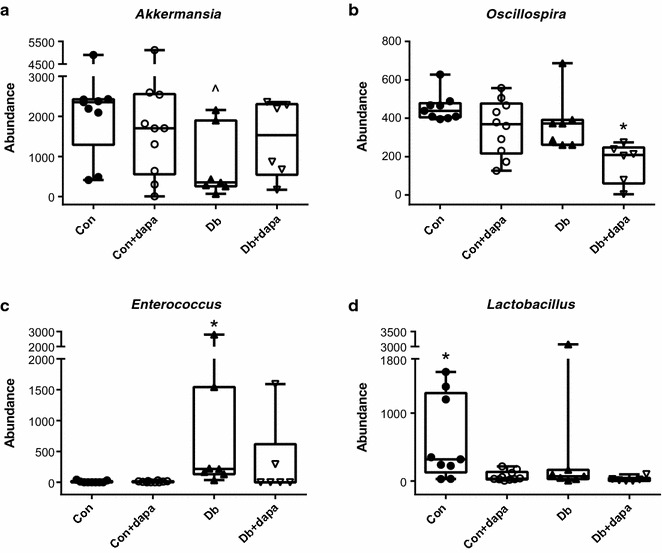
Species level characteristics of the microbiota. Abundance of **a** Akkermansia; **b** Oscillispira; **c** Enterococcus; **d** Lactobacillus. Box represents 25th–75th percentiles, median values are represented by boxplot internal line and ranges by whiskers; ^^^p < 0.05 vs Con; *p < 0.05 vs all other groups, ^#^p < 0.05 vs both Con groups; n = 6–10/group

**Fig. 8:**
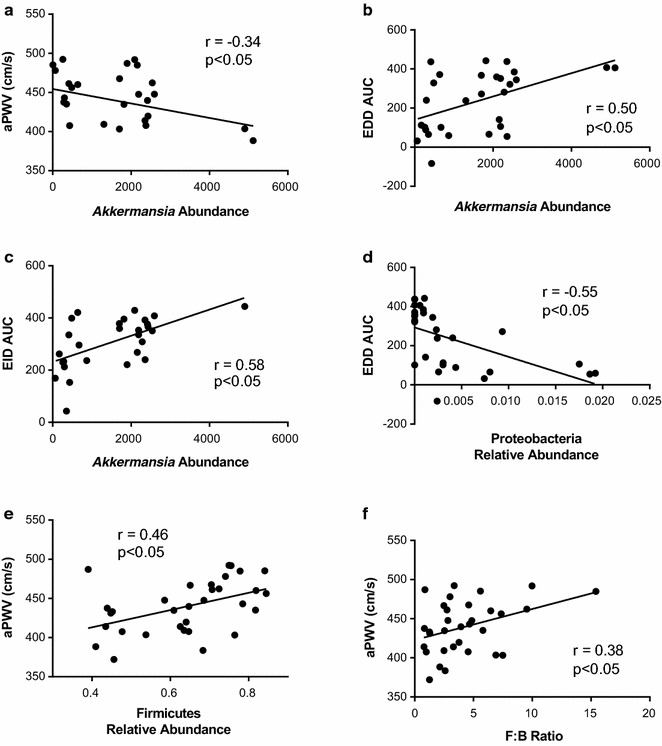
Correlations between microbiota and vascular outcomes. **a** aPWV and *Akkermansia* abundance; **b** EDD AUC and *Akkermansia* abundance; **c** EID AUC and *Akkermansia* abundance; **d** EDD AUC and Proteobacteria relative abundance; **e** aPWV and Firmicutes relative abundance; **f** aPWV and F:B ratio. r and p values are given following Pearson’s correlation test. n = 6–10/group

Using LEfSe, we found the abundance of several taxa differed among the experimental groups. For example, the composition of *Akkermansia muciniphila* was significantly decreased in Db compared to Con; *Oscillospira* was significantly reduced in the Db + dapa compared to all other groups; *Enterococcus* was significantly elevated in the Db group compared to all other groups; and *Lactobacillus* was significantly higher in the Con group compared to all other groups (Fig. 7a–d). Using Pearson’s correlation analysis, significant correlations were found between *A. muciniphila* and vascular function (Fig. 8a–c). Additionally, Proteobacteria, Firmicutes, and F:B ratio significantly correlated with vascular outcomes (Fig. 8d–f).

## Discussion

The primary findings of the present study were that diabetic mice treated with dapagliflozin for 8 weeks displayed significantly lower arterial stiffness, and improvements in endothelial dysfunction and vascular smooth muscle dysfunction compared to non-treated diabetic mice. These vascular improvements were accompanied by dramatic improvements in hyperglycemia and reductions in circulating markers of inflammation. Dapagliflozin also altered microbial diversity in diabetic animals but had little effect on control animals. Several specific taxa changes were also associated with dapagliflozin treatment in diabetic but not control animals, although the relevance of these changes to treatment efficacy remains unclear.

Arterial stiffness, as measured by aPWV, predicts cardiovascular events in patients with T2D and other populations at elevated CVD risk [41, 42]. We found that aPWV was significantly elevated in Db mice, but not Db mice consuming dapagliflozin. These results extend two published reports which found that SGLT2i reduced pulse pressure, an indirect marker of arterial stiffness [20, 43]. Solini found that acute (48 h) dapagliflozin treatment reduced PWV in patients with type 2 diabetes [18]. Therefore, the current results extend these existing studies by demonstrating that chronic SGLT2i reduces arterial stiffness in T2D mice.

We also found that EDD, another strong predictor of cardiovascular outcomes in patients with T2D [11, 42], was modestly and significantly improved in diabetic mice following dapagliflozin treatment. These data are consistent with those by Solini et al. [18] who found an improvement in flow mediated dilation (FMD) following acute (48 h) administration of dapagliflozin. Conversely, Shigiyama found no change in FMD following chronic (16 week) SGLT2i [19]. Lin et al. [38] reported modest improvements in EDD after 10 weeks of empagliflozin treatment in diabetic mice. EDD in the current study was measured in mesenteric arteries whereas Lin et al. reported changes in the aorta; thus, collectively, the two studies suggest that SGLT2i elicits favorable effects on endothelial function in both large elastic and smaller resistance arteries. These broad improvements across the vascular tree may enhance the clinical relevance given the relative importance of larger and smaller arteries on atherogenesis and blood pressure regulation. Despite significant improvements in EDD in Db + dapa compared to Db, values remained significantly lower compared to both control groups. The inability of SGLT2i to return EDD values closer to control levels may have been due to the incomplete resolution of hyperglycemia in the Db + dapa group. However, Lin et al. used a higher dose of SGLT2i and consequently observed a much greater reduction in blood glucose levels, but the improvements in EDD were still modest and similar to those in the current study. Another possibility is that other factors, either independent or downstream of hyperglycemia, may mediate a portion of the endothelial dysfunction but are not affected by SGLT2i.

Similar to aPWV and EDD, vascular smooth muscle dysfunction, as determined by reductions in EID, is common in T2D and predicts cardiovascular complications and mortality [16, 17]. We found that EID was significantly improved following SGLT2i. Several studies that examined the effects of SGLT2i on endothelial function did not report results on EID [18, 19]. Among the studies that did report EID, results have been somewhat conflicting. Han et al. [44] demonstrated that SGLT2i had negative, neutral, or positive effects on EID depending on the vessel type and the method of drug delivery. However, similar to the current study, Lin et al. found that EID was improved following SGLT2i in diabetic mice [38]. Given that dilation to ACh (EDD) ultimately requires smooth muscle cell relaxation, it is possible that the reductions in EDD and EID commonly observed in T2D mainly reflect dysfunction at the level of the smooth muscle cell rather than the endothelium. Regardless of the relative contribution of the two cell types, the current data indicate that dapagliflozin has pronounced effects on EID that may contribute to the reduction in CVD risk.

Compared to control mice, diabetic animals exhibited marked dysbiosis, which has been associated with numerous aspects of metabolic dysfunction [45, 46]. Treatment with dapagliflozin appeared to have little effect on the microbiota in control mice, but did cause subtle alterations in the richness and diversity of microbial communities in diabetic animals. Specifically, richness and diversity were reduced in Db + dapa, although the response seemed to be driven by minor differences across phyla rather than significant increases or decreases in specific bacterial taxa. Db + dapa-associated differences in Bacteroidetes and Proteobacteria might be partially responsible for global differences observed; however, there appear to be responder/non-responder animals, confounding biological interpretation of these changes. Overall, the ratio of Firmicutes:Bacteriodetes was reduced in Db + dapa animals compared to other groups. Higher levels of Bacteroidetes and a reduced Firmicutes:Bacteroidetes (F:B) ratio have been associated with a lean phenotype in previous studies [47]. In this case, the reduced F:B was not associated with a lean phenotype in the current study, as Db + dapa mice displayed significantly increased body weight compared to Db. Ussar et al. [48] recently suggested that these simplified metrics are not reliable predictors of metabolic outcomes, but rather are more reflective of varying environments interacting with genotypes. In either case the increase in body weight in this group may have been protective, as Db mice tend to lose weight as the severity of diabetes progresses, and medications that improve health outcomes in Db mice have been shown to preserve or increase body weight [37, 38].

Finally, at the species level, we also observed a trend for increased *Akkermansia muciniphila* in the Db + dapa group relative to Db. *A. muciniphila* has been shown to improve metabolic outcomes, including vascular function [49–51]. Furthermore, increases in *A. muciniphila* have recently been demonstrated following Metformin administration in diabetic individuals and mice, and have been suggested to mediate some of the protective effects of the drug [51, 52]. *Akkermansia* exclusively feeds off of the intestinal mucosa, therefore future studies are necessary to confirm whether SGLT2i administration is associated with an increase in *Akkermansia* and whether this might be the result of reductions in intestinal inflammation and an increase in protective mucosal secretions.

Chronic low-grade inflammation plays an important role in the cardio-metabolic consequences of gut dysbiosis [53]. Chronic inflammation is also an established mediator of vascular dysfunction [39, 40] and has been shown to explain much of the excess cardiovascular mortality in individuals with T2D [12]. We therefore determined several circulating markers of inflammation and found that all factors were markedly increased in Db mice and decreased by SGLT2i. These results are similar to those by Leng and colleagues [54], who reported that dapagliflozin reduced circulating levels of NLRP3, IL1β, and IL-18 in diabetic apoE^−/−^ mice. In type 2 diabetic mice, Tahara et al. [55] found that SGLT2i reduced serum levels of several of the same markers of inflammation determined in the current study, including IL-6 and MCP-1. Collectively, these data indicate that SGLT2i reduce systemic inflammation, although future studies in patients with T2D are necessary to determine the clinical relevance of these animal studies.

## Conclusions and limitations

Several limitations to the current study should be noted. First, although the db/db model used in the current study is the most widely used mouse model of T2D, and db/db mice do display gut dysbiosis [56], the monogenetic underpinnings of the model are not an exact representation of T2D in humans. Second, the current study was not designed to determine whether the improvements in vascular function were mediated by direct effects of SGLT2i on the vasculature or by indirect effects such as reductions in blood glucose. In vitro studies have indicated that SGLT inhibition may have direct effects within endothelial cells [57], but future studies are needed to directly address this issue. Similarly, although the vascular parameters measured in the current study are independent predictors of cardiovascular events in patients with T2D, we cannot state with any certainty that improvements in these parameters underlie the broader cardiovascular protection of SGLT2i observed in clinical trials. Lastly, fecal samples for gut microbiota analysis were only collected at the end of the study. Although consistencies in diet and housing conditions across all animals helped minimize fluctuations in gut microbiota composition, pre and post measures of the gut microbiota would have strengthened the study design.

In conclusion, the current data suggest that improvements in generalized vascular dysfunction may underlie the beneficial cardiovascular effects of SGLT2i. Furthermore, in diabetic animals, SGLT2i was associated with subtle alterations in microbial richness and diversity which appear to be mainly due to the accumulation of non-significant variations across multiple taxa. The extent to which these alterations mediate the beneficial effects of SGLT2i on vascular function are unclear. Future studies utilizing co-administration of antibiotics or intravenous administration of SGLT2i are necessary to comprehensively address this issue.

## Abbreviations

T2D: type 2 diabetes
CVD: cardiovascular disease
SGLT2i: sodium glucose cotransporter 2 inhibitors
Db: male diabetic mice
Con: male control mice
dapa: dapagliflozin (Farxiga)
aPWV: aortic pulse wave velocity
AUC: area under the curve
EID: endothelium-independent dilation
EDD: endothelium-dependent dilation
PVAT: perivascular adipose tissue
Ach: acetylcholine
PE: phenylephrine
SNP: sodium nitroprusside
